# *In vitro* assessment of berberine-loaded carboxymethyl chitosan hydrogel: A promising antimicrobial candidate for *S. aureus*-induced bovine mastitis treatment

**DOI:** 10.1371/journal.pone.0326574

**Published:** 2025-06-27

**Authors:** Wenjia Wang, Huimin Huang, Yuetong Gu, Jiahang Li, Yian Tao, Gengshao Ma, Zhihai Shi

**Affiliations:** 1 College of Veterinary Medicine, Henan University of Animal Husbandry and Economy, Zhengzhou, China; 2 Institute of Animal Husbandry and Veterinary Medicine, Henan Academy of Agricultural Sciences, Zhengzhou, China; Annamalai University, INDIA

## Abstract

Bovine mastitis poses significant challenges to the global dairy industry, leading to substantial economic losses and public health concerns. *Staphylococcus aureus*, a prevalent causative agent of bovine mastitis, depends on effective adhesion and biofilm formation to establish infections. Berberine (BER), a naturally occurring phytochemical, demonstrates broad-spectrum antibacterial activity but suffers from poor bioavailability. This study developed a composite berberine-carboxymethyl chitosan/sodium alginate hydrogel to address these limitations. The hydrogel was characterized using scanning electron microscopy, Fourier-transform infrared spectroscopy, and X-ray diffraction. *In vitro* assessments revealed that the BER hydrogel eradicated *S. aureus* biofilms (42% eradication at 156.26 μg/mL), inhibited bacterial adhesion, and reduced inflammatory cytokines (IL-6 and TNF-α) in *S. aureus*-infected MAC-T cells, with compliant biosafety biocompatibility (hemolysis rate <5%) and sustained drug release (100% over 6 h), though pH-dependent release kinetics necessitate microenvironment-specific formulation refinement. In conclusion, the BER hydrogel represents a potential therapeutic candidate for *S. aureus*-induced bovine mastitis.

## Introduction

Bovine mastitis, a prevalent and costly disease in the global dairy industry, significantly impacts milk quality and quantity, leading to economic losses estimated at approximately 147 $ per cow [1]. This condition poses a major challenge to both the dairy industry and public health. *Staphylococcus aureus* (*S. aureus*), a leading cause of mastitis in dairy cows worldwide, is particularly detrimental, as it reduces key milk components such as lactose, fat, protein, and milk urea nitrogen [2]. It can persist on the udder and is easily transmitted from infected to uninfected teats during milking. Antibiotics are considered the first choice for the treatment of bovine mastitis. However, the problem of antibiotic residue and antimicrobial resistance, in addition to the impact of antibiotic abuse on public health, leads to many restrictions on uncontrolled antibiotic therapy in the dairy sector worldwide. Therefore, the development of alternative therapeutic strategies that present novel avenues against *S. aureus* infections are increasingly demanded and gaining more and more attention.

In recent decades, phytochemicals have garnered significant attention due to their bioactive properties and their ability to combat bacterial infections without inducing resistance, offering a promising antibiotic-free alternative for treating *S. aureus* infections. Berberine (BER), a natural isoquinoline alkaloid and the primary component of *Coptis chinensis*, has demonstrated a strong safety profile in clinical applications [3]. It exhibits a wide range of biological activities, including antibacterial, anti-inflammatory, antioxidant, antitumor, and antidiabetic properties [4,5]. Notably, BER shows potent antibacterial activity against *S. aureus*, including the Methicillin-Resistant *S. aureus* [6], with minimal side effects, making it a more suitable for clinical use compared to traditional antibiotics. Common side effects of BER consumption include gastrointestinal symptoms, such as constipation and diarrhea. However, its therapeutic potential is hindered by poor water solubility, low intestinal absorption, rapid metabolism, and extremely low oral bioavailability [7]. To address these limitations, the development of an effective delivery system is essential to enhance BER’s pharmacological efficacy and overcome its current drawbacks.

Hydrogels are versatile materials widely used in biomedicine and biotechnology, offering a promising platform for antibacterial therapy by enhancing drug bioavailability, adhesion, controlled release, and overall therapeutic efficacy [8,9]. Natural polysaccharide-based hydrogels, in particularly, exhibit excellent biocompatibility, self-healing capabilities, and adhesion properties, making them highly suitable for biomedical applications [10,11]. Chitosan (CS), a positively charged polymer, can electrostatically interact with the lipopolysaccharide membranes of bacteria, facilitating its adhesion to bacterial surfaces. This property makes it widely utilized in the formulation of antibacterial hydrogels [12]. Carboxymethyl chitosan (CMCS), a water-soluble derivative of CS with carboxymethyl functional groups, is renowned for its antimicrobial properties, non-toxicity, biodegradability, and biocompatibility, making it a preferred material for hydrogel preparation [13]. Sodium alginate (SA), a natural copolymer composed of β-D mannuronic acid and α-L glucuronic acid, is characterized by its high water absorption capacity and ease of gel formation [14]. It has been reported that due to its negative charge, SA can serve as an effective drug delivery system for antimicrobial peptides, thereby enhancing their antimicrobial activity [15]. The abundant carboxyl and hydroxyl groups in SA’s glucuronic acid structure enable it to crosslink with the amino groups in CMCS under specific conditions, resulting in hydrogels with tailored properties and applications. Previous studies have demonstrated that intramammary infusion of CS-based hydrogels can reduce the incidence of bovine mastitis by promoting immune cell migration and accelerating involution after drying-off [16]. Additionally, self-assembled CS/SA nanogels have been shown to enhance the therapeutic efficacy of Tilmicosin against *S. aureus* induced bovine mastitis [17]. However, to date, there is limited research on the preparation and properties of CMCS/SA hydrogels loaded with BER for the treatment for bovine mastitis. Therefore, this study aims to develop a BER-loaded CMCS/SA hydrogel and evaluate its efficacy in a *S. aureus*-induced mastitis model using the MAC-T cell line (bovine mammary epithelial cells).

**Fig 1:**
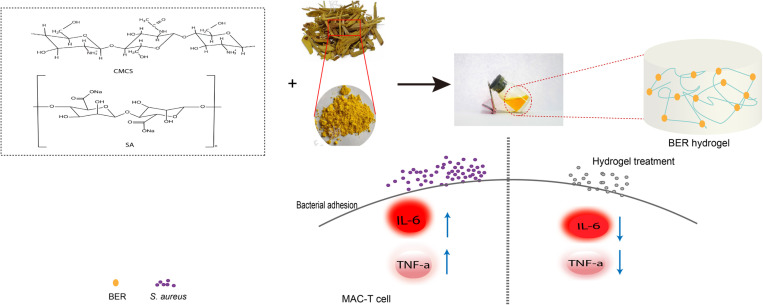
Schematic illustration of BER hydrogel preparation and its anti-inflammatory effects on *S. aureus*-infected MAC-T cells.

In this study, a CMCS/SA hydrogel loaded with BER was successfully prepared. The hydrogel’s efficacy against *S. aureus* was comprehensively evaluated, demonstrating significant antibacterial and anti-inflammatory properties. The results revealed that the BER-loaded hydrogel effectively alleviated inflammatory damage in *S. aureus*-infected MAC-T cells, a model of bovine mammary epithelial cells. In summary, these findings highlight the potential of BER-loaded CMCS/SA hydrogel as a promising therapeutic agent for the treatment of bovine mastitis, paving the way for its future application in the dairy industry (Fig 1).

## Materials and methods

### Preparation and physicochemical characterization of BER hydrogel

#### Materials.

CMCS (MW 20–30 kDa, degree of carboxylation~80%), hydroxyethyl cellulose (HEC, 5500–6500 mPas) and SA were purchased from Solarbio Science & Technology Co., Ltd., (Beijing, CHN). Berberine extract (BER, S27357, HPLC ≥ 95%) was purchased from Yuanye Bio-Technology Co., Ltd., (Shanghai, China). *S. aureus* ATCC 29213 was obtained from the Guangzhou Institute of Microbiology (Guangzhou, China).

#### Preparation of BER hydrogel.

The BER solution with a concentration of 2.5 mg/mL was prepared by dissolving 12.5 mg of BER in 0.5 mL of dimethyl sulfoxide under complete dissolution, followed by dilution to a final volume of 5 mL with deionized water. The CMCS/SA hydrogel was prepared through stirring until the hydrogel formed. Briefly, 1 g of CMCS, 1 g of SA and 1 g of HEC were dissolved in 100 mL of deionized water, and then stirred at 60°C and allowed to react for a few minutes. Then, 1 mL of the above BER solution was mixed thoroughly with 1 mL of CMCS/SA hydrogel (1: 1 ratio), and the BER hydrogel was obtained.

#### Physicochemical characterization.

Fourier-transform infrared (FTIR) spectroscopy was conducted on the BER extract, CMCS/SA hydrogel, and BER hydrogel over a range of 4500-400 cm^-1^ using a FTIR spectrometer (FTIR-7600, Lambda, Australia). X-ray diffraction (XRD) patterns of the BER extract, CMCS/SA hydrogel, and BER hydrogel were obtained using an X-ray diffractometer (XRD-7000, Shimadzu, Japan) at 40 kV, with a scan range of 10 to 80 degrees. The morphology of the BER hydrogel was observed using scanning electron microscopy (SEM; S-3000N, Hitachi, Japan). For SEM imaging, the BER hydrogel samples were first frozen in liquid nitrogen slush (- 210°C) for 2 min, then fractured at -130°C, and finally etched at -75°C for 30 min. The cross-sectional surface of the hydrogel was coated with gold under vacuum by an ion sputter before observation [18].

The swelling ratio (SR) of the BER hydrogel was measured as previously described [19]. Briefly, the hydrogel was dried and weighed (W_0_), then rehydrated in 5 mL of phosphate-buffered saline (PBS, pH 7.4) at 37°C for various time intervals (1, 2, 3, 5, 8, 11, 20, 40 h, etc.). After each time interval, the swollen hydrogel was removed from the PBS, and excess surface moisture was blotted with filter paper. The hydrogel was then weighed again (W_s_). SR was calculated using the following equation:


$$\begin{array}{c} \text{SR = \textbackslash{} (Ws - W0)\textbackslash{} /\textbackslash{} W0}\times 100\% \end{array}$$

The release behavior of BER from the BER hydrogel was evaluated using the basket method by simulating body fluids and performing periodic sampling to measure the concentration of the drug released [20]. The environments used were simulated acidic fluid with a pH of 3.0, simulated physiological fluid with a pH of 7.4 (which suitable for *S. aureus* growth), and simulated basic liquid with a pH of 8.9, respectively. A dialysis membrane containing 20 mL of PBS buffer solution and 5 mg of BER hydrogel was placed in a beaker filled with PBS buffer at 37°C, with stirring at 100 rpm. At specific time intervals (2 min, 5 min, 10 min, 15 min, 0.5 h, 1 h, 2 h, 4 h, and 6 h), 1 mL of the supernatant was sampled from the beaker, and the beaker was replenished with 1 mL of fresh PBS buffer. The absorbance of the sample was measured at 340 nm. The concentration of BER was determined from the standard curve of a BER solution prepared in PBS buffer at concentration of 5 μg/mL^-1^–50 μg/mL^-1^. The corresponding regression equation was (μg/mL^-1^) y = 0.0495x + 0.0823, R^2^ = 0.9982, where x is the drug concentration, y is the absorbance of the drug. A cumulative release curve was subsequently plotted. To further understand the drug delivery mechanism, various models such as the zero-order, first-order, Higuchi, and Korsmeyer-Peppas models were used to kinetically fit the drug release curves. The release index *n* and kinetic rate constant *k* were calculated for each model by linear regression analysis. The accuracy of fitness was evaluated through the correlation coefficient R^2^.

#### Hemolysis assessment.

The blood compatibility of the BER hydrogel was evaluated *in vitro* using a hemolysis assay. BER hydrogel were prepared at concentrations of 2.5, 0.25, and 0.025 mg/mL with PBS. A 2% suspension of bovine red blood cell was mixed with the hydrogel samples and incubated at 37°C for 6 h. After incubation, the absorbance of the supernatant was measured at 540 nm using a spectrophotometer. For controls, bovine blood samples were mixed with deionized water (positive control) and PBS (negative control). The hemolysis rate was calculated using the following formula:


$$\begin{array}{c} \text{Hemolysis ratio}\;(\%)\;=\;\text{(ODs - ODn)/\textbackslash{} (ODp - ODn)}\;\times \;100\% \end{array}$$

where ODn, ODp, and ODs represent the optical density values of the negative control, positive control, and sample, respectively.

### Antibacterial activity of BER hydrogel against *S. aureus*

The minimum inhibitory concentration (MIC) of BER hydrogel against *S. aureus* ATCC 29213 was determined via broth microdilution in Trypticase Soy Broth. Bacterial suspensions (~10⁸ CFU/mL) were exposed to serial dilutions of BER hydrogel (19.53-1250 μg/mL) and incubated at 37°C for 24 h. The MIC was defined as the lowest concentration inhibiting visible bacterial growth. For SEM analysis, *S. aureus* suspensions (10⁸ CFU/mL) were treated with or without BER hydrogel at 37°C for 24 h. The samples were then centrifuged at 2,500 × g for 5 min, and the supernatant was discarded. Bacterial samples were subsequently fixed with glutaraldehyde and osmium tetroxide, dehydrated using an ethanol series, and subjected to critical-point drying. After gold sputter-coating, morphological changes were observed using SEM (Hitachi SU8100, Japan).

The antibiofilm efficacy of BER hydrogel was assessed against *S. aureus* biofilms using crystal violet staining. Bacterial suspensions (10⁸ CFU/mL) were incubated for 24 h to form biofilms, followed by treatment with BER hydrogel at 0.5 × MIC, 1 × MIC, and 2 × MIC concentrations. After fixation (methanol) and staining (0.1% crystal violet), bound dye was solubilized with glacial acetic acid, and absorbance (570 nm) was measured. The biofilm eradication rate was calculated as:


$$\begin{array}{c} \text{Eradication\textbackslash{} ratio}\;(\%)\;=\;\text{(ODn - ODs)/\textbackslash{} (ODn)}\;\times \;100\% \end{array}$$

where ODn and ODs represent the optical density values of the negative control and sample, respectively.

### Anti-inflammatory injury of BER hydrogel in *S. aureus* -induced mastitis MAC-T cell model

#### Cell line culture.

The MAC-T cell line (bovine mammary epithelial cells), derived from bovine udder tissue, was purchased from Keycell Biotechnology Co., Ltd. (No. QS-Q008). Cells were cultured in complete growth medium containing Dulbecco’s Modified Eagle Medium (DMEM)/Ham’s F12 (No. 11965092; Gibco, San Francisco, CA, USA) supplemented with 10% fetal bovine serum (FBS; No. 10091, Gibco) and 1% penicillin-streptomycin (No. SC118, Seven, Beijing, China). For viability assays, cells were seeded into 96-well plates, while gene expression and cytokine secretion analyses were performed using cells plated in 6-well plates with 2 × 10^6^ cells per well. Cultures were maintained at 37°C in a humidified 5% CO₂ atmosphere, with medium replenishment every 2–3 days. Cells were sub-cultured upon reaching 80–90% confluence, typically every 3–4 days.

#### Cytotoxicity assessment.

MAC-T cells (six replicates per treatment) were seeded into 96-well plates (2 × 10^4^ cells per well), and incubated at 37°C in 5% CO_2_ atmosphere for 24 h. After removing the culture medium, cells were treated with DMEM/F12 medium (FBS-free) containing various concentrations of BER hydrogel (78.13, 39.06, 19.53, and 9.77 μg/mL) and incubated for 24 h. Subsequently, 10 μL of CCK-8 reagent (CCK-8; No. SC119, Seven, Beijing, China) was added to each well, and cells were incubated for an additional 3 h. Absorbance was measured at 450 nm using a microplate reader (BioTek Synergy H1, Winooski, VT, USA).

#### Anti-inflammatory and anti-infection evaluation.

Based on the results of the cytotoxicity assessment, a non-cytotoxic concentration of 10 μg/mL BER hydrogel was selected for further studies. Cells were divided into six groups and given the following treatments: 1) CON group: DMEM only; 2) BER hydrogel group: DMEM + 10 μg/mL BER hydrogel; 3) CMCS/SA hydrogel group: DMEM +10 μg/mL CMCS/SA hydrogel; 4) *S. aureus* group: DMEM + *S. aureus* (MOI = 100); 5) BER hydrogel-*S. aureus* group: DMEM + 10 μg/mL BER hydrogel + *S. aureus* (MOI = 100); 6) CMCS/SA hydrogel-*S. aureus* group: DMEM + 10 μg/mL CMCS/SA hydrogel + *S. aureus* (MOI = 100). All treatments were performed in serum-free DMEM for 6 h. The post-incubation procedures systematically assess treatment effects through: 1) Cytokine quantification (ELISA) in centrifuged supernatants to evaluate anti-inflammatory response. 2) Supernatants containing *S. aureus* were serially diluted, plated on TSA, and incubated at 37°C for 24 h to quantify CFU, for bacterial invasion. 3) qPCR analysis of cytokine-related genes in cell pellets to probe molecular mechanisms. All steps included triplicate biological replicates and untreated controls, ensuring methodological rigor.

**Cell Viability:** MAC-T cells (six replicates per treatment) were seeded into 96-well plates (2 × 10^4^ cells per well), and incubated at 37°C in 5% CO_2_ atmosphere for 24 h. After removing the culture medium, cells were treated with DMEM/F12 medium (FBS-free) containing various concentrations of BER hydrogel and incubated for 24 h. Subsequently, 10 μL of CCK-8 reagent was added to each well, and cells were incubated for an additional 3 h. Absorbance was measured at 450 nm using a microplate reader (BioTek Synergy H1, Winooski, VT, USA).

#### Cytokine secretion.

Interleukin-6 (IL-6) and tumor necrosis factor-α (TNF-α) levels in cell supernatant were quantified using bovine-specific enzyme-linked immunosorbent assay (ELISA) kits (Fine Biotech, Wuhan, China) following the manufacturer’s protocol. Briefly, 100 μL of supernatant or standard was added to antibody-coated wells and incubated for 2 h at 37°C. After washing, horseradish peroxidase (HRP)-conjugated detection antibodies were added, followed by tetramethylbenzidine (TMB) substrate. Reactions were stopped with 2 M H₂SO₄, and absorbance was measured at 450 nm. Cytokine concentrations were calculated from standard curves and expressed as picograms per milliliter (pg/mL). All samples were analyzed in triplicate.

#### Cell invasion assay.

MAC-T cells were seeded into 6-well plates (2 × 10^6^ cells/well), and incubated for 24 h at 37°C under 5% CO_2_. The culture medium was then removed; then, the medium (FBS-free) was supplemented with or without CMCS/SA hydrogel, BER hydrogel and *S. aureus*. After 6 h of treatment, cells were washed three times with PBS and detached using 0.25% trypsin-EDTA at 37°C for 5 min. Using a 1 mL pipette, the contents of each well were gently pipetted multiple times to ensure proper mixing. The cell suspension was serially diluted (10-fold). Aliquots (100 μL) were plated on TSA and incubated at 37°C for 36 h. CFU were enumerated to quantify intracellular bacterial invasion.

#### Gene expression.

The levels of IL-6 and TNF-α in the cells were quantified. Total RNA was extracted from MAC-T cells across various treatment groups using the TRIzol reagent following the manufacturer’s instructions. RNA concentration was determined with a NanoDrop 2000 (Thermo Fischer Scientific, Waltham, MA, USA), and RNA purity was assessed by measuring the OD260/OD280 ratio, which was expected to be between 1.80 and 2.10 to ensure adequate purity. The integrity of the RNA samples was further evaluated and confirmed via agarose gel electrophoresis. First-strand cDNA was synthesized using the MightyScript First Strand cDNA Synthesis Master Mix (Sangon Biotech, Shanghai, China). The synthesized cDNA was subsequently utilized for quantitative real-time PCR (qRT-PCR) employing the AceQ SYBR® qPCR Master Mix (SEVEN, Beijing, China). Genes expression levels were measured using the LightCycler 480 system (Roche, Basel, Switzerland). Data were analyzed using the 2^−ΔΔCt^ method, with expression levels normalized to the housekeeping gene GAPDH. The Primers used for qRT-PCR are listed in Table 1.

**Table 1:** Related primer sequences in this study.

Gene	Primer	Primer Sequence (5′-3′)	Size(bp)	Accession numbers
IL-6	F	CCACCCCAGGCAGACTACTTC	63	NM_173923.2
	R	CCATGCGCTTAATGAGAGCTT		
TNF-α	F	CCACGTTGTAGCCGACATCA	132	AF348421.1
	R	ATGAGGTAAAGCCCGTCAGC		
GAPDH	F	GGGTCATCATCTCTGCACCT	176	NM_001034034.2
	R	GGTCATAAGTCCCTCCACGA		

#### Statistical analysis.

Data analysis was performed using the Statistical Package within GraphPad Prism 9.0 software. Independent t-tests were conducted for assessment of cell viability, and one-way ANOVA was used for the analysis of the other data. Statistical significance was determined by p-values, with p < 0.01 indicating an extremely significant difference and p < 0.05 indicating a significant difference among groups.

## Results

### Preparation of the BER hydrogel

**Fig 2:**
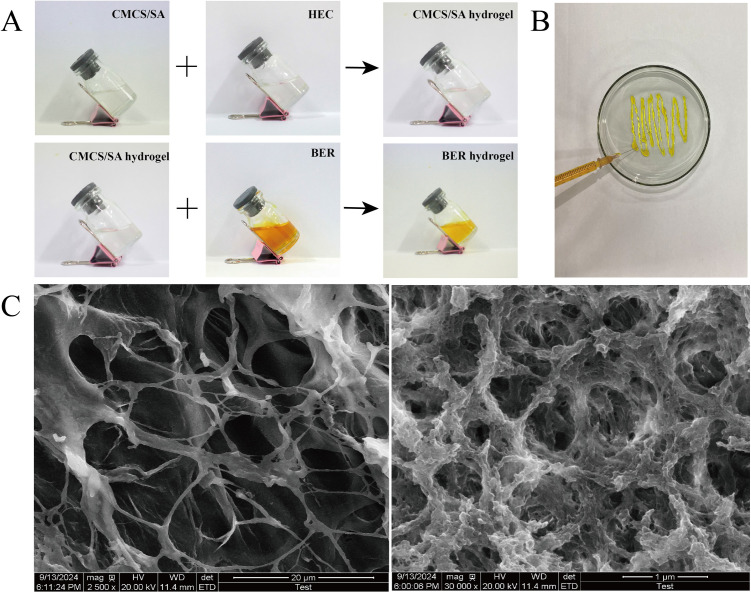
Preparation and SEM images of the BER hydrogel. **(A)**. The formation process of BER hydrogel. **(B)**. injectability of the hydrogel. **(C)**. SEM images of the cross sections of BER hydrogel.

The preparation procedure for the BER hydrogel is illustrated in Fig 2A. The color of the hydrogel changed from colorless to a translucent yellow upon the addition of BER. The prepared hydrogel demonstrates injectable properties (Fig 2B) and suitable for use on irregularly shaped wounds. The cross-sectional morphology of BER hydrogel is depicted in Fig 2C. SEM revealed that the BER hydrogel exhibited a three-dimensional continuous porous network structure. The network structure is crucial for the hydrogel’s properties, including swelling, compressibility, cell adhesion, and drug diffusion.

### Physicochemical characterization of the BER hydrogel

**Fig 3:**
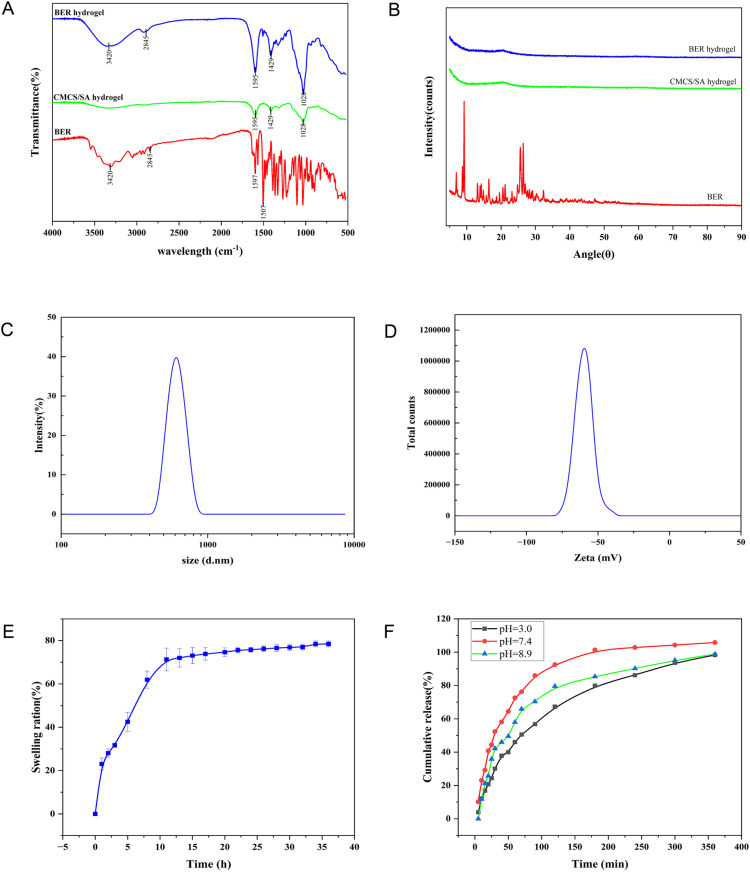
Physicochemical characterization of the BER hydrogel. **(A)**. FTIR spectra, **(B)**. XRD patterns of the BER extract, CMCS/SA hydrogel and BER hydrogel. The pore sizes (C) and the Zeta potential (D) of BER hydrogel. The swelling ratio (E) of BER hydrogel were determined in PBS (pH = 7.4) at 37°C; and cumulative release of BER from the BER hydrogel (F) in PBS (pH = 3.0), PBS (pH = 7.4), and PBS (pH = 8.9) within 6 h at 37°C.

FTIR analysis was conducted to characterize the chemical profiles of BER extract, CMCS/SA hydrogel, and BER hydrogel, with spectral signatures systematically compared in Fig 3A. In the spectrum of the CMCS/SA hydrogel, the stretching vibration absorption of the -OH band belonging to CMCS was observed at 1,429 cm^-1^, while the peak at 1595 cm^-1^, corresponding to the -COOH band, was associated with SA. For the BER extract, in addition to the stretching vibration of -OH at 3420 cm^-1^ and the vibration of -CH at 2845 cm^-1^, the absorption bands at 1597 cm^-1^ and 1507 cm^-1^ were linked to the vibrations of the benzene ring skeleton [21]. The spectra of the BER hydrogel exhibited characteristic peaks of CMCS, SA and BER, indicating that BER has been successfully incorporated into the CMCS/SA system without disrupting the organic functional groups on its surface.

XRD analysis of BER extract, CMCS/SA hydrogel, and BER hydrogel within the 2θ-range of 10–80 deg. revealed distinct structural features (Fig 3B). Both CMCS/SA and BER hydrogels exhibited a prominent amorphous diffraction peak at 2θ = 22 deg., attributed to the planar stacking of polymer chains formed by the copolymerization of CMCS and hydroxyethyl cellulose. Notably, the presence of crystalline BER-specific peaks in the hydrogel’s XRD pattern confirmed successful encapsulation of BER within the CMCS/SA matrix.

SEM analysis indicated that the BER hydrogel possessed a porous architecture with pore sizes ranging from 500 to 600 nm (Fig 3C). While these values reflect surface morphology rather than true hydrogel mesh size, the submicron-scale porosity facilitates permeation through physiological barriers while maintaining structural integrity. Zeta potential measurements demonstrated a surface charge of −60 mV for the BER hydrogel (Fig 3D), indicative of strong electrostatic repulsion and colloidal stability. Swelling studies revealed rapid hydrogel hydration, with SR increasing sharply from 1 to 11 hours (reaching values of approximately 68% at 11h), followed by a plateau phase (Fig 3E, S1 Dataset). The *in vitro* release of BER from the BER hydrogel was evaluated at an acidic solution with a pH of 3.0, physiological fluid with a pH of 7.4 (which suitable for *S. aureus* growth), and a basic solution with a pH of 8.9, respectively. The cumulative release rate of BER at solution with a pH of 7.4 was the highest compared with the acidic solution and basic solution (Fig 3F, S2 Dataset). The drug release kinetics displayed a biphasic profile: an initial burst release (50% within the first 0.5 hours) attributed to surface-associated BER, followed by a sustained diffusion-driven release (100%% over 6 hours) (Fig 3F). As presented in Table 2, the comparative analysis of model fitting revealed superior performance of the first-order kinetic model in describing the drug release profile of BER hydrogel, as evidenced by its higher R² value compared to alternative models. These findings demonstrate that the release behavior of BER from the hydrogel matrix follows first-order kinetics, aligning with fundamental characteristics of sustained-release formulations. The observed kinetic pattern suggests that the incorporated drug molecules are primarily distributed along the inner architecture of the hydrogel matrix rather than being uniformly dispersed. This mechanistic interpretation of drug-matrix interaction shows strong agreement with recent findings reported by Tong et al. [22] in similar hydrogel delivery systems.

**Table 2:** Fitted parameters obtained using models for the drug release data.

Samples	Zero-order		First-order		Higuchi model		Korsmeyer-Peppas model		
	k	R^2^	k	R^2^	k	R^2^	k	n	R^2^
pH = 3.0	0.2537	0.8660	0.0109	0.9924	0.5074	0.8660	5.648	0.4966	0.9738
pH = 7.4	0.2348	0.6738	0.0212	0.9926	0.4695	0.6738	15.54	0.3462	0.9054
pH = 8.9	0.2400	0.7286	0.0162	0.9866	0.4799	0.7286	9.425	0.4152	0.9143

### BER hydrogel effectively inhibited *S. aureus* and eradicated biofilm

**Fig 4:**
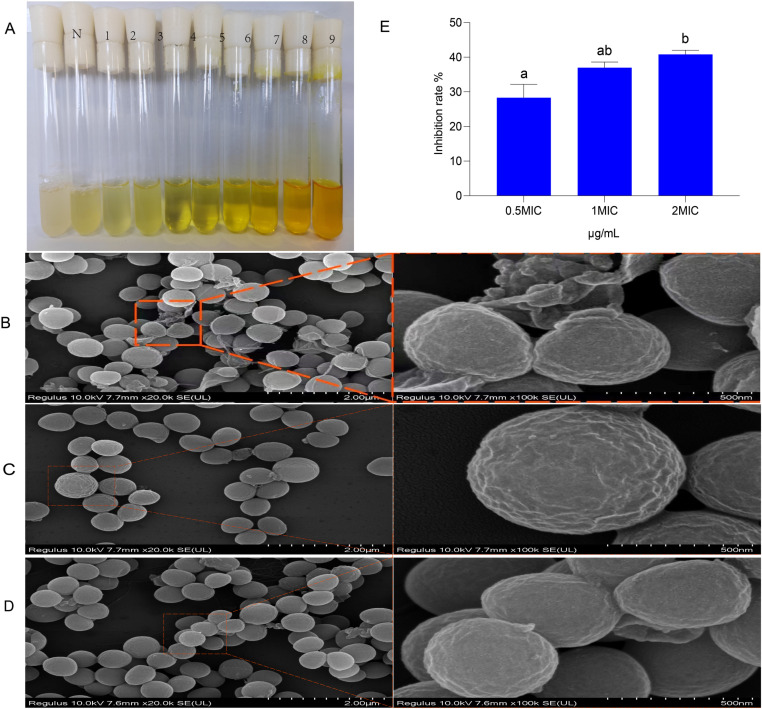
Effects of BER hydrogel on cell morphology and its biofilm of *S. aureus.* **(A)**. The MIC of BER hydrogel, the columns 1-9 were 9.77, 19.53, 39.06, 78.13, 156.25, 312.5, 625, 1250 and 2,500 μg/mL of BER hydrogel, respectively, while the column N (from left to right) (negative control) *S. aureus* was used. **(B)**. SEM analysis of cell morphology of *S. aureus* treated with 0.5 MIC BER hydrogel for 24 **h. (C)**. SEM analysis of cell morphology of normal *S. aureus*. **(D)**. SEM analysis of cell morphology of *S. aureus* treated with CMCS/SA hydrogel. **(E)**The biofilm eradication rate of BER hydrogel in the concentration range of 0.5-2MIC (n = 6/group). Data were analyzed by one-way ANOVA. p-value indicates the difference among the groups; p < 0.05 indicates a significant difference. Groups with different letters had significant differences (p < 0.05).

The BER hydrogel demonstrated a MIC of 78.13 μg/mL against *S. aureus* (Fig. 4A). To further elucidate its antibacterial mechanism, SEM was used to examine bacterial morphological alterations following treatment with the BER hydrogel at 0.5 × MIC. *S. aureus* adhered to the hydrogel and exhibited abnormal morphology (Fig 4B, S1 Fig). In contrast, the untreated group and CMCS/SA hydrogel group displayed smooth, round bacterial surfaces, while the BER hydrogel group showed membrane depression and wrinkling, indicating that the bacterial membrane was damaged by the cationic CS and BER components (Fig 4C-4D, S2 and S3 Figs).

The crystalline violet staining method was used for the quantitative analysis of bacterial biofilms. The biofilm eradication rate increased significantly with increasing concentrations of the BER hydrogel (Fig 4E, S3 Dataset). At a concentration of 156.26 μg/mL, the biofilm eradication rate of BER hydrogel was approximately 42%.

### BER hydrogel alleviated inflammatory injury in *S. aureus* -induced mastitis MAC-T cell model

**Fig 5:**
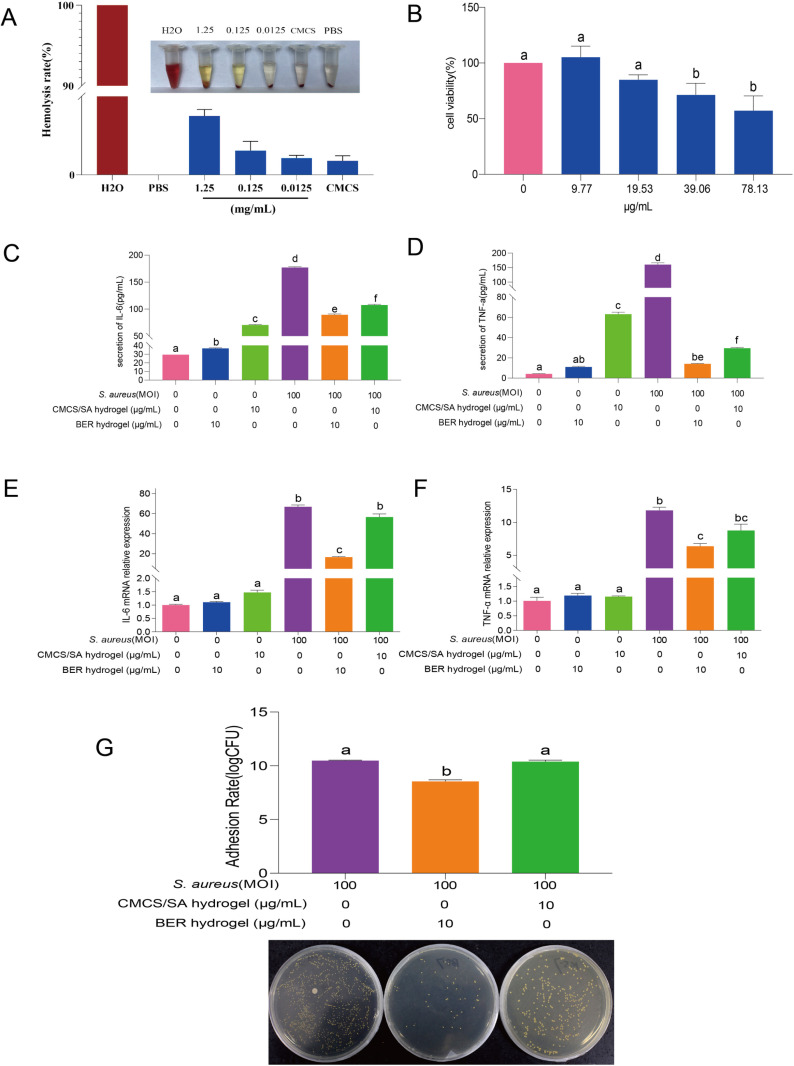
The biocompatibility and anti-inflammatory injury of BER hydrogel in *S. aureus* -induced mastitis MAC-T cell model. **(A)**. The hemolysis test of BER hydrogel. **(B)**. The cell viability of MAC-T cells stimulated by 9.77-78.13 μg/mL BER hydrogel for 24 h. **(C, D)**. The effects of BER hydrogel on the secretion of IL-6 and TNF-α in *S. aureus*- stimulated cells. **(E, F)**. The gene expression of IL-6 and TNF-α in *S. aureus* stimulated cells. **(G)**. The effects of BER hydrogel on the adhesion rate of *S. aureus*. Independent t-tests were conducted for data analysis of cell viability, other data were analyzed by one-way ANOVA. p -value indicates the difference among the groups; p < 0.01 indicates an extremely significant difference. Groups with different letters had significant differences (p < 0.01).

The biocompatibility of BER hydrogel was assessed through a hemolysis assay. The results of the bovine blood compatibility test showed that after adding PBS solution or BER hydrogel, aggregated bovine red blood cells were observed at the bottom of the sample, while the positive control group (sterile deionized water) displayed bright red coloration (Fig 5A, S4 Dataset). Quantitative analysis revealed that the hemolysis rate of BER hydrogel was below 5%, indicating its excellent hemocompatibility. The viability of the MAC-T cells treated with < 19.53 μg/mL of BER hydrogel did not differ significantly (p > 0.05) from the 0 μg/mL group after 24 h of treatment (Fig 5B). Based on these results, 10 μg/mL BER hydrogel was selected for further analysis. Compared with the CON group, secretion of IL-6 and TNF-α in the *S. aureus* group was significantly elevated (p < 0.01) (Fig 5C-5D); however, BER hydrogel treatment moderated this increase (p < 0.01). S. aureus stimulation also significantly upregulated the gene expression of IL-6 and TNF-α (p < 0.01), while BER hydrogel supplementation alleviated these effects (p < 0.01) (Fig 5E-5F).

Recent studies have highlighted the critical roles of adhesion, invasion, and evasion in the inflammation process induced by *S. aureus* [23]. Since adhesion is the initial interaction between *S. aureus* and host cells, we investigated the effect of BER hydrogel on the adhesion of *S. aureus* to MAC-T cells. The adhesion rate of *S. aureus* to MAC-T cells was significantly lower in BER hydrogel group compared with the *S. aureus* group (p < 0.01) (Fig 5G), suggesting that BER hydrogel effectively reduces the adhesion of *S. aureus* to MAC-T cells.

## Discussion

Bovine mastitis, an inflammatory condition of the udder caused by physical damage, chemical irritation, or pathogens infection [24], poses a significant challenge to dairy production. *S. aureus*, a predominant pathogen in intramammary infections [25], is particularly detrimental due to its ability to evade host defenses and drastically reduce milk yield compared to other pathogens. Prevention of *S. aureus* infection of the mammary gland remains challenging. Conventional antibiotic therapies face limitations due to rising antimicrobial resistance and regulatory constraints. Hydrogels, as versatile biomaterials, have emerged as promising tools in biomedical engineering [26]. Although hydrogel systems face limitations including compromised viscoelastic mechanical properties under dynamic loading, mismatched bioerosion kinetics relative to tissue regeneration timelines, and batch-to-batch variability complicating clinical translation, targeted applications demonstrate therapeutic promise. Previous studies have demonstrated that intramammary hydrogel infusion during the dry period enhances resistance to infection [16]. BER, a widely used traditional Chinese medicine ingredient with strong antibacterial properties, is an excellent choice for enhancing the antibacterial ability of hydrogel [21]. In this study, we developed a hybrid hydrogel incorporating BER, a natural compound with potent antibacterial properties, to address the limitations of traditional treatments. Our findings reveal that the BER hydrogel effectively combats *S. aureus* biofilms *in vitro* and mitigates inflammatory responses in MAC-T cell model, offering a novel strategy for the prevention and treatment of bovine mastitis.

The anti-infective treatment is crucial for the teat or udder tissue healing. The BER hydrogel’s injectability, demonstrated by its smooth passage through a 23G needle, enables minimally invasive delivery to deep or irregular lesions, reducing reliance on surgical interventions. This property is particularly advantageous for localized treatment of the teat canal, thereby reducing the need for invasive surgery [27]. The BER hydrogel’s three-dimensional porous network, formed through self-assembly of two oppositely charged polysaccharides, CMCS (positive charge) and SA (negative charge), facilitates efficient drug loading and controlled release. The interconnected pores (500–600 nm) promote gas exchange at wound sites, accelerating tissue repair while maintaining structural integrity. These features underscore BER hydrogel’s potential as a dual-function platform for antibacterial and regenerative therapies.

Biofilm formation by *S. aureus* enhances its resistance to antibiotics and host immune responses [28]. Hydrogel can serve as an antibiotic vehicle for topical treatment of bacterial biofilms [29]. Our study demonstrated concentration-dependent biofilm eradication by the BER hydrogel, achieving 42% clearance at 156.26 μg/mL. This investigation also demonstrates marked adhesion of *S. aureus* to the BER-encapsulated hydrogel matrix, concomitant with distinct morphological aberrations characterized by cell wall invagination and irregular septation patterns. CS, as the main carrier for BER hydrogel, which is positively charged, can electrostatically interact with the lipopolysaccharide membrane of bacteria, allowing it to adhere to the bacteria and making it widely used in the preparation of antibacterial hydrogels [12]. The hydrogel’s cationic CMCS component electrostatically interacts with bacterial membranes, promoting adhesion and membrane disruption. BER release kinetics revealed an initial burst phase (50% within 0.5 hours) followed by sustained diffusion (100% cumulative release over 6 hours). This biphasic profile ensures rapid pathogen suppression and prolonged antibacterial activity. The synergy between CMCS-mediated adhesion and BER’s bactericidal action likely underpins the hydrogel’s superior performance.

Biocompatibility is critical for therapeutic hydrogels [30]. Our findings revealed that the BER hydrogel exhibited exceptional hemocompatibility (hemolysis rate < 5%) and no cytotoxicity to MAC-T cells at concentrations ≤ 19.53 μg/mL. These findings demonstrate that BER hydrogel has good cytocompatibility and align with clinical safety requirements for topical applications.

Inflammation in the mammary gland is a response of the host immune system to invading microorganisms, and its intensity varies depending on the type of infectious agent. *S. aureus*-associated bovine mastitis exhibits characteristically insidious inflammatory kinetics, frequently manifesting as subclinical infections with attenuated proinflammatory cytokine cascades and histopathologically occult tissue infiltration [31]. The epithelial cells lining the inner surface of the mammary gland recognize the pathogens responsible for mastitis, triggering the release of proinflammatory signaling molecules that are crucial for both local and systemic immune reactions in the animal [32]. It is well-studied that proinflammatory cytokines, such as TNF-α and IL-6, play a central role in the inflammatory response associated with bovine mastitis. Additionally, previous research has suggested that serum cytokines could serve as indirect markers in control strategies for managing bovine mastitis [33]. In this study, the expression and cytokine secretion of inflammatory mediators (TNF-α and IL-6) in MAC-T cells induced by *S. aureus* were significantly increased. However, following treatment with BER hydrogel, the levels of these inflammatory cytokines were notably reduced. Additionally, BER hydrogel was found to decrease the adhesion rate of *S. aureus* to MAC-T cells. Therefore, we speculate that the decreased adhesion rate of *S. aureus* to MAC-T cell by BER hydrogel may act as a kind of ‘anti-inflammatory pathway’. To sum up, these results demonstrate that BER hydrogel showed excellent anti-inflammatory properties from the inflammation of MAC-T cells infected by *S. aureus.* These results will help facilitate the application of BER hydrogel in bovine mastitis.

## Conclusions

This study establishes the critical necessity of BER-encapsulated hydrogels as multifunctional therapeutic platforms for combating *S. aureus* infections in dairy production systems. The developed hydrogel demonstrates potent antimicrobial efficacy (MIC = 78.13 μg/mL) and biofilm eradication capacity (42% reduction at 2 × MIC), while concurrently mitigating *S. aureus*-induced inflammatory cascades in MAC-T cells. Such dual-action systems address urgent industry demands for non-antibiotic alternatives that can reduce mastitis-associated milk losses while maintaining milk quality standards. The pH-responsive drug release mechanism further suggests adaptability to mammary gland microenvironmental conditions, positioning this technology as a paradigm-shifting approach for sustainable dairy herd management.

## Supporting information

S1 Dataset The swelling ratio of BER hydrogel. The data were obtained by weighing the hydrogels at different time points in PBS (pH = 7.4) at 37°C.
(XLSX)

S2 Dataset The cumulative release of BER from the BER hydrogel. The data were calculated based the corresponding regression equation, y = 0.0495x + 0.0823, R2 = 0.9982, where x is the drug concentration, y is the absorbance of the drug.
(XLSX)

S3 Dataset The biofilm eradication rate of BER hydrogel in the concentration range of 0.5–2MIC. The absorbance (570 nm) was measured.
(XLSX)

S4 Dataset The hemolysis test of BER hydrogel. The absorbance (540 nm) was measured.
(XLSX)

S1 Fig Cell morphology of *S. aureus* treated with 0.5 MIC BER hydrogel. SEM analysis of cell morphology of *S. aureus* treated with 0.5 MIC BER hydrogel for 24 h.
(DOCX)

S2 Fig Cell morphology of normal *S. aureus.* SEM analysis of cell morphology of normal *S. aureus*.
(DOCX)

S3 Fig Cell morphology of *S. aureus* treated with CMCS/SA hydrogel. SEM analysis of cell morphology of *S. aureus* treated with CMCS/SA hydrogel for 24 h.
(DOCX)
