# Supplementary figures and images for: In vitro assessment of berberine-loaded carboxymethyl chitosan hydrogel: A promising antimicrobial candidate for S. aureus-induced bovine mastitis treatment
Source: PLoS One. 2025 Jun 27;20(6):e0326574. doi: 10.1371/journal.pone.0326574 (PMC12204576; doi:10.1371/journal.pone.0326574)

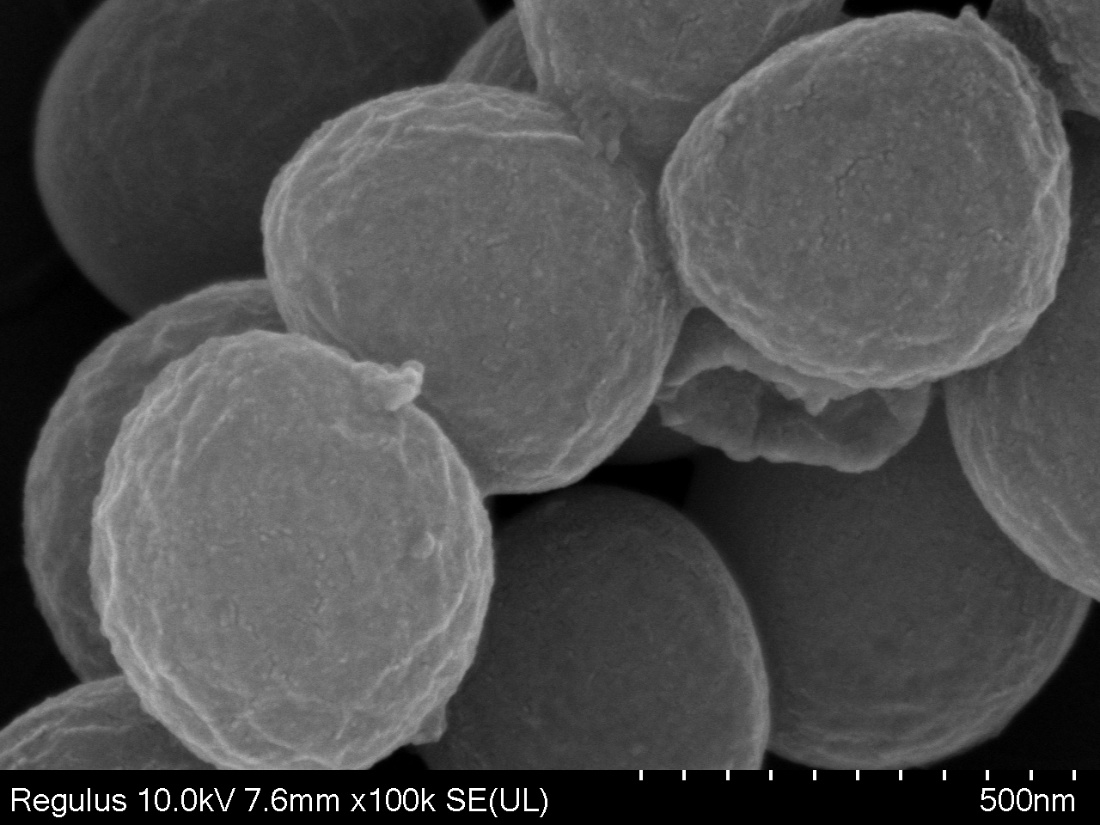

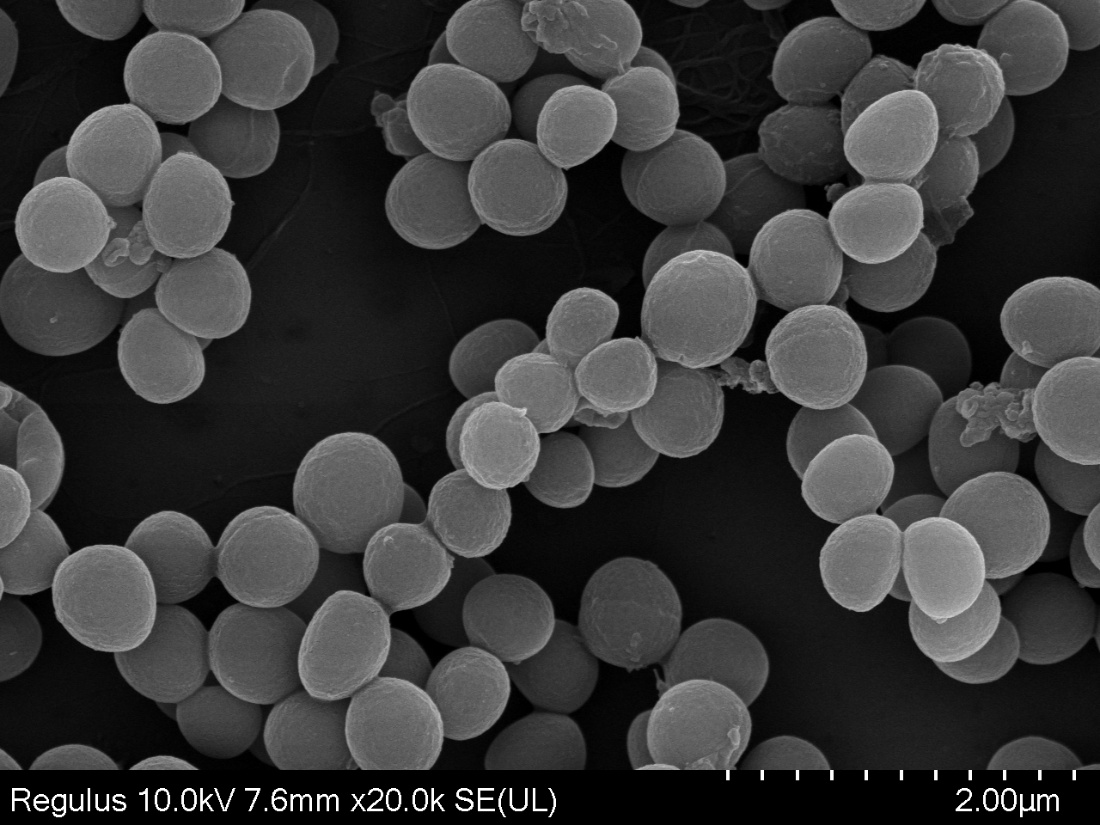


**S2 Fig. Cell morphology of normal *S. aureus*.** SEM analysis of cell morphology of normal *S. aureus*.

Supplement: S2 Fig — SEM analysis of cell morphology of normal S. aureus. (DOCX) [file pone.0326574.s006.docx]
