# Supplementary material for: In vitro assessment of berberine-loaded carboxymethyl chitosan hydrogel: A promising antimicrobial candidate for S. aureus-induced bovine mastitis treatment
Source: PLoS One. 2025 Jun 27;20(6):e0326574. doi: 10.1371/journal.pone.0326574 (PMC12204576; doi:10.1371/journal.pone.0326574)
Supplement: S3 Fig — SEM analysis of cell morphology of S. aureus treated with CMCS/SA hydrogel for 24 h. (DOCX) [file pone.0326574.s007.docx]

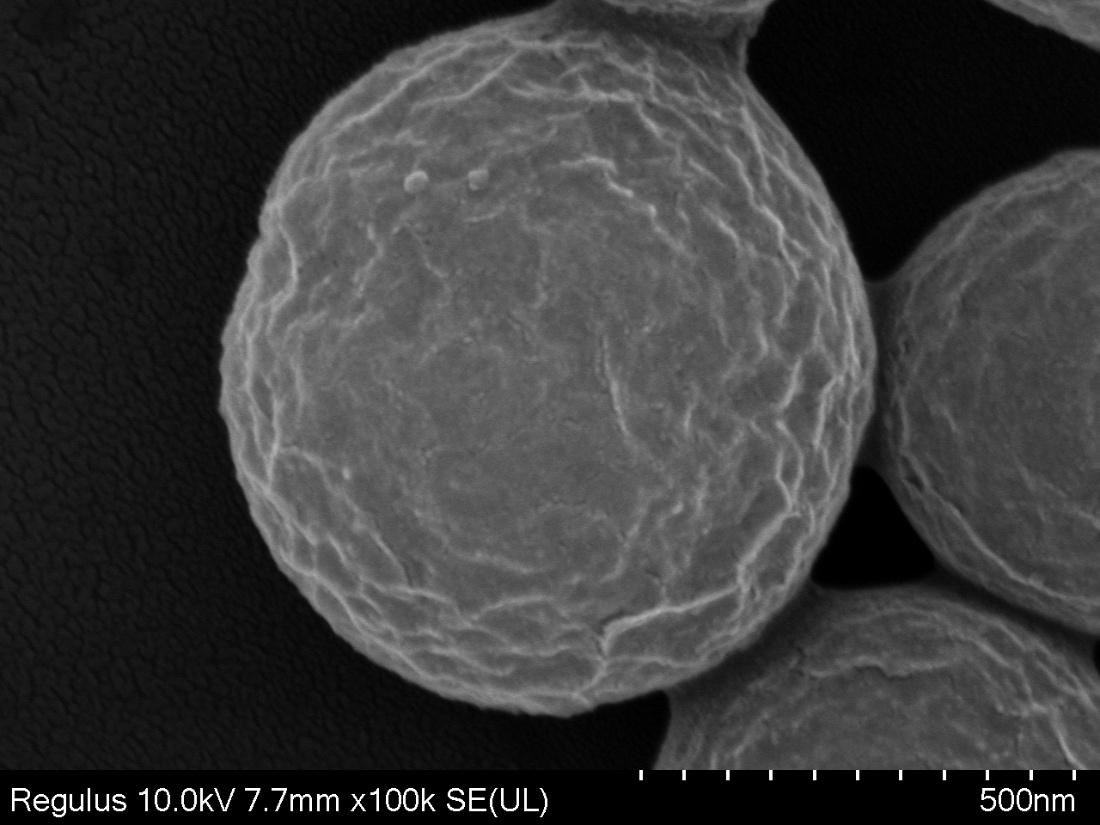

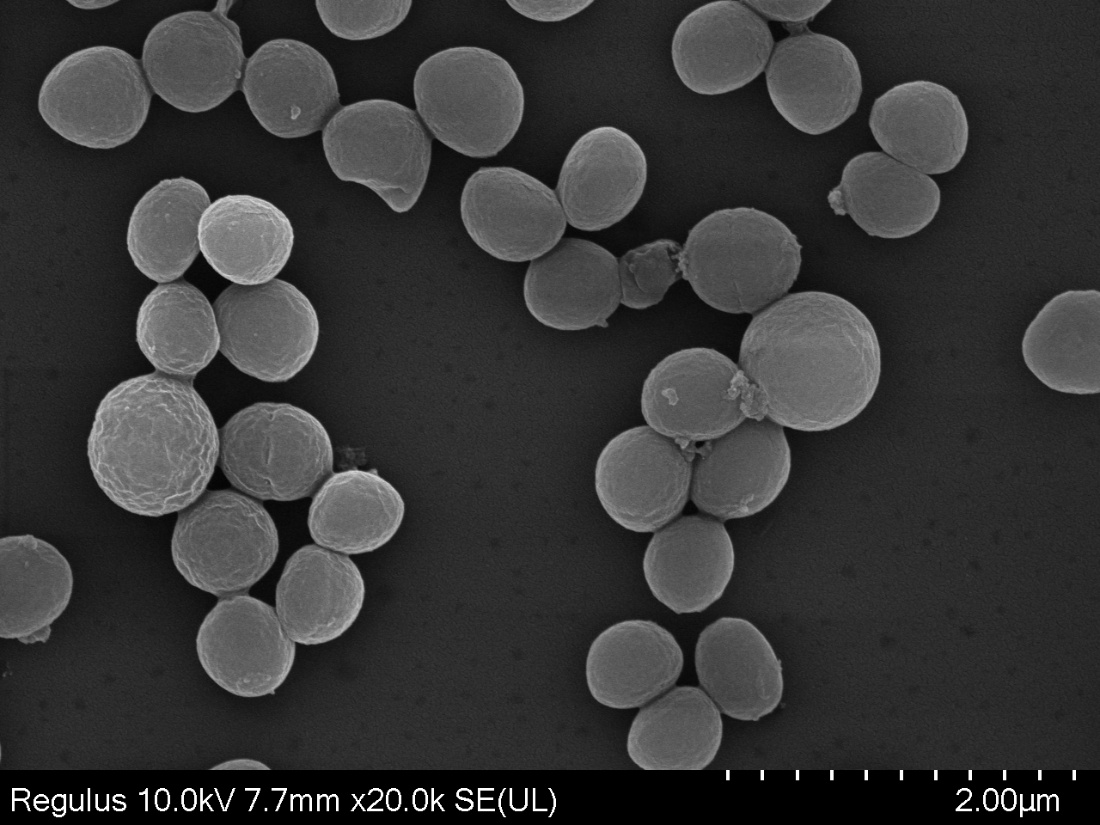


**S3 Fig. Cell morphology of *S. aureus* treated with CMCS/SA hydrogel.** SEM analysis of cell morphology of *S. aureus* treated with CMCS/SA hydrogel for 24 h.
